# Dynamic nested hierarchies: self-evolving machine learning architectures for lifelong learning

**DOI:** 10.3389/frai.2026.1804338

**Published:** 2026-05-14

**Authors:** Akbar Anbar Jafari, Cagri Ozcinar, Gholamreza Anbarjafari

**Affiliations:** 1 Institute of Technology, University of Tartu, Tartu, Estonia; 2 Estonian Business School, Tallinn, Estonia; 3 3S Holding, Tartu, Estonia

**Keywords:** catastrophic forgetting, class-incremental learning, continual learning, dynamic nested hierarchies, lifelong learning, nested optimization, neurogenesis, neuroplasticity-inspired AI

## Abstract

Contemporary machine learning models, including large language models, exhibit remarkable capabilities in static tasks yet falter in non-stationary environments due to rigid architectures that hinder continual adaptation and lifelong learning. Building upon the nested learning (NL) paradigm, which decomposes models into multi-level optimization problems with fixed update frequencies, this work proposes Dynamic Nested Hierarchies (DNH) as an extension enabling autonomous structural adaptation. Unlike static nested learning where hierarchy depth and update frequencies are fixed at initialization, DNH introduces three biologically-grounded mechanisms: (1) level addition triggered by meta-loss thresholds, analogous to adult neurogenesis in the hippocampal dentate gyrus; (2) level pruning based on gradient contribution, analogous to synaptic elimination; and (3) frequency modulation driven by local surprise signals, analogous to neural oscillation adaptation. We provide explicit mappings between these mechanisms and neuroplasticity processes, moving beyond superficial analogy to principled design. Through rigorous mathematical formulations, we prove convergence bounds of *O*(1/*T* + δ^2^) in non-stationary environments, expressivity improvements bounded by ϵ ≤ *O*(1/*L*_*t*_) + γδ, and sublinear regret $O\left(\sqrt{T}\right)$ compared to static architectures' linear regret. Empirical evaluations on language modeling, continual learning benchmarks—including comparisons with Elastic Weight Consolidation (EWC) and Synaptic Intelligence (SI) as well as modern methods (DER++, MEMO) on Split ImageNet, CLEAR-100, and CORe50—and long-context reasoning validate the theoretical advantages. Comprehensive ablation studies verify the contribution of each component, including the Self-Modifying Memory (SMM) module and Evolutionary Adam (EAdam) optimizer. We provide detailed computational cost analysis and parameter trajectory visualizations demonstrating bounded growth through self-regulating pruning.

## 1 Introduction

Advancements in deep learning have propelled machine learning models to unprecedented capabilities in domains such as natural language processing, computer vision, and multimodal understanding (Radford, 2009; Vaswani et al., 2017; Jafari et al., 2025a). However, these models predominantly rely on static architectures that excel in fixed-distribution tasks but exhibit profound limitations in non-stationary environments, where data distributions evolve over time. This rigidity manifests as catastrophic forgetting, suboptimal generalization to out-of-distribution data, and an inability to continually acquire new knowledge without retraining, akin to anterograde amnesia in neurological contexts (Behrouz et al., 2025). The core issue stems from the fixed parameterization and update mechanisms in traditional deep networks, which fail to accommodate dynamic context flows and multi-time-scale adaptations necessary for lifelong learning.

Recent works have highlighted these challenges through innovative architectural designs and theoretical frameworks. For instance, the Nested Learning (NL) paradigm decomposes deep learning models into multi-level nested optimization problems, each with distinct update frequencies, revealing that optimizers like Adam and SGD with momentum function as associative memory modules compressing gradient flows (Behrouz et al., 2025). While NL provides a mathematically rigorous reformulation of sequence models and optimizers, enabling higher-order in-context learning via components such as deep optimizers and self-modifying titans, its static hierarchy limits adaptability in continual setups. Similarly, hybrid frameworks integrating graph-enhanced structures with large language models (LLMs), such as GEYOLO-AHC, address scalability in real-time object detection by adaptively conducting heat for feature propagation, yet they remain constrained to specific tasks without general lifelong evolution (Jafari and Anbarjafari, 2025). Furthermore, mathematical modeling of AI singularity emphasizes recursive self-improvement bounds and control mechanisms for responsible AI, underscoring the need for dynamic systems that mitigate unbounded growth while ensuring ethical deployment (Hoffmann, 2023; Ishizaki and Sugiyama, 2025; Jafari et al., 2025b, 2026).

Contemporary efforts in dynamic neural architectures further illuminate the problem. EvoNet introduces self-evolving networks that autonomously adjust structures during training via genetic-inspired mutations, demonstrating improved robustness in reinforcement learning tasks (Tutuncuoglu et al., 2025). Growing Neural Networks leverage gradient-based expansion to dynamically add layers, achieving sublinear regret in non-stationary optimization (Papadigenopoulos et al., 2022). Dynamic Retrieval-Augmented Expert Networks incorporate mixture-of-experts routing for lifelong language tasks, reducing forgetting through selective parameter updates (Long et al., 2025). Neuroplasticity-inspired models, such as those mimicking synaptic consolidation, enable continual adaptation by emulating human brain mechanisms like sharp-wave ripples for memory replay (Xie et al., 2025; Zhang et al., 2025; Li et al., 2025). Early foundational work by Ring (1994) introduced CHILD networks with context-dependent incremental structure, establishing neurogenesis-inspired growth as a viable approach to continual learning. More recently, Draelos et al. (2017) demonstrated neurogenesis deep learning with explicit neuron addition mechanisms. Despite these advances, existing approaches often lack a unified framework for autonomous hierarchy evolution, leading to inefficiencies in handling volatile contexts and distribution shifts.

### 1.1 Positioning against dynamic architecture methods

Several prior works enable structural adaptation in neural networks, but differ fundamentally from DNH. Network Architecture Search (NAS) (Zoph and Le, 2017) searches for optimal static architectures offline, whereas DNH adapts online during training and inference; NAS does not support continual adaptation. Progressive Neural Networks (Rusu et al., 2016) add lateral connections for new tasks but never prune, leading to unbounded growth, whereas DNH's pruning mechanism maintains efficiency. Dynamic Network Expansion methods (Yoon et al., 2018) add neurons or layers heuristically, while DNH uses gradient-based meta-optimization with theoretical convergence guarantees (Theorem 1). EvoNet (Tutuncuoglu et al., 2025) uses genetic mutations for structure evolution, which is stochastic and lacks convergence guarantees, whereas DNH's meta-gradient approach is principled and provably convergent. Ring's CHILD architecture (Ring, 1994) pioneered continual structural growth but lacked principled pruning; DNH combines growth with gradient-based pruning under theoretical guarantees. The key innovation of DNH is combining (1) nested optimization structure from NL, (2) gradient-based meta-optimization for structural decisions, and (3) frequency modulation for multi-timescale adaptation—a combination absent from prior work.

To address the limitations of static architectures, this work proposes Dynamic Nested Hierarchies (DNH), an extension of NL that endows models with self-evolution capabilities. DNH represents architectures as time-varying directed acyclic graphs where levels, dependencies, and frequencies adapt via meta-optimization, drawing from neuroplasticity to facilitate lifelong learning without fixed constraints. Mathematically, DNH optimizes a meta-loss incorporating distribution shifts, ensuring convergence in non-stationary regimes as proven through regret bounds and expressivity analyses.

#### 1.1.1 Contributions

This work makes the following distinct contributions beyond the static NL framework of (Behrouz et al., 2025):

1. **Dynamic hierarchy evolution:** We formalize DNH as a time-varying directed acyclic graph (DAG) with autonomous level addition and pruning, whereas NL uses fixed static hierarchies. This is the core architectural contribution enabling lifelong learning.
2. **Biologically-grounded mechanisms:** We provide explicit mappings between DNH operations and neuroplasticity: level addition corresponds to adult neurogenesis (not LTP, which governs weight initialization), level pruning to synaptic elimination, and frequency modulation to neural oscillation adaptation (Section 2.3).
3. **Theoretical guarantees:** We prove convergence, expressivity, and regret bounds specific to dynamic hierarchies under distribution shift (Theorems 1–3), extending beyond NL's static analysis.
4. **Comprehensive evaluation:** We compare against continual learning baselines (EWC, SI) and modern methods (DER++, Co2L, CLS-ER, MEMO) on both classical and modern benchmarks (Split ImageNet, CLEAR-100, CORe50), and provide ablation studies for all core components (SMM, EAdam, meta-optimization framework). We include detailed computational cost analysis and parameter growth trajectories.

The remainder of this paper is structured as follows. Section 2 formalizes DNH, detailing its advantages over static NL and adaptation mechanisms. Section 3 presents self-evolving DNH models with architectural designs and integration examples. Section 4 provides mathematical proofs, including convergence and stability analyses. Section 5 evaluates DNH empirically on benchmarks, with ablation studies and application implications. Section 6 concludes with future directions.

## 2 Dynamic nested hierarchies

In this section, we introduce Dynamic Nested Hierarchies (DNH), a novel extension of the NL paradigm presented in Behrouz et al. (2025). While NL decomposes machine learning models into static multi-level optimization problems with fixed update frequencies, DNH enables models to autonomously adapt the number of levels, their nesting structure, and update frequencies during training or inference. This dynamism addresses the limitations of static architectures in handling non-stationary data distributions, drawing inspiration from neuroplasticity in the human brain, where synaptic strengths and neural pathways evolve in response to new experiences (Dayan and Cohen, 2011). We formalize DNH mathematically, demonstrating how it enhances model expressivity and facilitates lifelong learning.

**Terminology and definitions**. Throughout this paper, we use the following consistent terminology:

- **Level vs. module:** We use “level” to refer to the hierarchical position ℓ ∈ {1, …, *L*_*t*_} and “module” to refer to the computational unit $M_{t}^{\left(\ell \right)}$ at that level. A module is a memory mapping; a level is its position in the hierarchy.
- **Memory module:** An associative memory $M_{t}^{\left(\ell \right)}:K_{t}^{\left(\ell \right)}\to V_{t}^{\left(\ell \right)}$ mapping query keys to values. Following attention terminology, we denote queries as **q**, keys as **k**, and values as **v**.
- **Distribution shift:** A change in the data-generating distribution *p*_*t*_(**x**) over time, quantified by Δ_*t*_ = *D*_KL_(*p*_*t*_||*p*_*t*−1_) or total variation distance.
- **Non-stationary environment:** A setting where $p_{t}\left(\text{x}\right)\neq p_{t^{\prime }}\left(\text{x}\right)$ for some *t* ≠ *t*′.

### 2.1 Limitations of static nested learning

The NL framework represents a model as a set of nested optimization problems, each with distinct update frequencies *f*_*A*_ for component *A*, ordered by the relation *A* ≻ *B* if *f*_*A*_ > *f*_*B*_ or if *A*'s computation at time *t* depends on *B*'s state at *t* (as defined in Behrouz et al., 2025). However, this structure is predefined and static, fixed at initialization. For a neural learning module with *L* levels, the overall optimization is expressed as:


$$\begin{array}{l} \theta^{*}=\arg\underset{\theta^{\left(1\right)}}{\min}L^{\left(1\right)}\left(\theta^{\left(1\right)};D\right), \end{array}$$ (1)

where θ^(1)^ parameterizes the outermost level, and inner levels ℓ = 2, …, *L* solve sub-problems:


$$\begin{array}{l} \theta^{\left(\ell \right)}=\arg\underset{\theta^{\left(\ell \right)}}{\min}{\tilde{L}}^{\left(\ell \right)}\left(\theta^{\left(\ell \right)};\theta^{\left(\ell -1\right)},{\text{c}}^{\left(\ell \right)}\right), \end{array}$$ (2)

with **c**^(ℓ)^ denoting the context flow at level ℓ, such as gradients or tokens.

This static hierarchy struggles in non-stationary environments, where data distributions shift over time, as seen in continual learning tasks (Parisi and Kanan, 2019; Parisi and Lomonaco, 2020). For instance, LLMs under NL exhibit “anterograde amnesia,” limiting adaptation beyond fixed pre-training phases or context windows (Behrouz et al., 2025). Mathematically, the expressivity is bounded by the initial depth *L* and frequencies ${\left\{f^{\left(\ell \right)}\right\}}_{\ell =1}^{L}$, which do not evolve, leading to suboptimal convergence rates in varying regimes. Empirical evidence from NL's HOPE module shows improved but still limited performance in long-context reasoning due to rigid level definitions.

### 2.2 Formal definition of dynamic nested hierarchies

To overcome these limitations, we define a Dynamic Nested Hierarchy as a time-varying directed acyclic graph (DAG) $G_{t}=\left(V_{t},E_{t}\right)$, where vertices $V_{t}={\left\{M_{t}^{\left(\ell \right)}\right\}}_{\ell =1}^{L_{t}}$ represent memory modules (associative memories as per Definition 1 in Behrouz et al., 2025) at time *t*, and edges $E_{t}$ encode nesting dependencies. The number of levels *L*_*t*_ is dynamic, and each module $M_{t}^{\left(\ell \right)}$ has an adaptable update frequency $f_{t}^{\left(\ell \right)}\in {\mathbb{R}}^{+}$.

The state of the hierarchy at time *t* is governed by a meta-optimization process:


$$\begin{array}{l} G_{t+1}=\arg\underset{G}{\min}L_{\text{meta}}\left(G;G_{t},{\text{x}}_{t},\Delta_{t}\right), \end{array}$$ (3)

where $L_{\text{meta}}$ is a meta-loss measuring adaptation efficacy, **x**_*t*_ is the current input, and Δ_*t*_ quantifies distribution shift (e.g., via Kullback-Leibler divergence *D*_KL_(*p*(**x**_*t*_)||*p*(**x**_*t*−1_))). Intuitively, this meta-optimization updates the hierarchy structure $G_{t+1}$ to minimize adaptation cost given the current input **x**_*t*_ and measured distribution shift Δ_*t*_, balancing task performance with structural stability.

Each module $M_{t}^{\left(\ell \right)}:K_{t}^{\left(\ell \right)}\to V_{t}^{\left(\ell \right)}$ maps keys to values in its local context flow, optimized as:


$$\begin{array}{l} M_{t}^{\left(\ell \right)*}=\arg\underset{M^{\left(\ell \right)}}{\min}{\tilde{L}}^{\left(\ell \right)}\left(M^{\left(\ell \right)};M_{t}^{\left(\ell -1\right)},{\text{c}}_{t}^{\left(\ell \right)}\right), \end{array}$$ (4)

with dependencies following $E_{t}$.

The frequency $f_{t}^{\left(\ell \right)}$ is updated via a gradient-based rule inspired by momentum in NL optimizers:


$$\begin{array}{l} f_{t+1}^{\left(\ell \right)}=f_{t}^{\left(\ell \right)}+\eta_{f}\nabla_{f_{t}^{\left(\ell \right)}}L_{\text{meta}}+m_{t+1}^{\left(\ell \right)}, \end{array}$$ (5)

where $m_{t+1}^{\left(\ell \right)}=\beta m_{t}^{\left(\ell \right)}+\left(1-\beta \right)\nabla_{f_{t}^{\left(\ell \right)}}L_{\text{meta}}$, and η_*f*_, β are hyperparameters. This frequency update rule increases *f*^(ℓ)^ when the meta-loss gradient is positive (indicating the current frequency is suboptimal) and incorporates momentum *m*^(ℓ)^ for smooth adaptation, analogous to Adam's momentum in parameter optimization. This allows frequencies to increase for rapidly changing contexts (e.g., high surprise signals) or decrease for stable ones, mimicking brain wave adaptations (delta to gamma frequencies) (Buzsáki and Wang, 2012).

### 2.3 Neurobiological grounding of DNH mechanisms

The term “neuroplasticity” in neuroscience refers to the brain's ability to reorganize itself by forming new neural connections and eliminating unused ones (Dayan and Cohen, 2011). We now establish precise correspondences between DNH mechanisms and neurobiological processes, moving beyond analogy to principled design. Note that while biological neuroplasticity typically operates at the neuron and synapse level, DNH implements analogous principles at the module and layer level, which we term “component-level plasticity.”

#### 2.3.1 Level addition and adult neurogenesis

In biological neural networks, adult neurogenesis—the generation of new neurons in the hippocampal dentate gyrus—enables the brain to expand its representational capacity in response to novel experiences (Aimone et al., 2014; Draelos et al., 2017). Our level addition mechanism directly mirrors this: when meta-loss exceeds threshold τ (indicating insufficient representational capacity), a new module $M^{\left(L_{t}+1\right)}$ is instantiated, analogous to new neurons being integrated into existing circuits. This mapping is more appropriate than Long-Term Potentiation (LTP), as LTP strengthens existing connections rather than creating new computational units. Ring's CHILD architecture (Ring, 1994) pioneered this neurogenesis-inspired approach; DNH extends it with principled meta-optimization.

#### 2.3.2 Hebbian initialization and LTP

Long-Term Potentiation (LTP) strengthens synaptic connections when pre- and post-synaptic neurons fire together repeatedly (Do, 1949). In DNH, LTP corresponds to the Hebbian initialization of newly added modules:


$$\begin{array}{l} \theta_{t+1}^{\left(L_{t}+1\right)}=\theta_{t}^{\left(L_{t}\right)}+\alpha {\text{c}}_{t}^{\left(L_{t}\right)}{\left({\text{c}}_{t}^{\left(L_{t}\right)}\right)}^{⊤}, \end{array}$$ (6)

where the outer product **c**(**c**)^⊤^ implements Hebbian correlation learning to initialize new module weights based on existing context flow patterns.

#### 2.3.3 Level pruning and synaptic elimination

During development, the brain undergoes synaptic pruning, eliminating underutilized connections to improve efficiency (Dayan and Cohen, 2011). DNH's pruning criterion $\|\nabla_{\theta_{t}^{\left(\ell \right)}}L^{\left(1\right)}\|<\epsilon$ identifies modules with minimal contribution to learning, analogous to synapses with low activity being eliminated. This prevents overfitting and reduces computational overhead, mirroring the brain's optimization of neural circuits.

#### 2.3.4 Frequency modulation and neural oscillations

Brain activity exhibits oscillations at different frequencies: delta waves (0.5–4 Hz) during deep sleep, theta waves (4–8 Hz) during memory encoding, and gamma waves (30–100 Hz) during active cognition (Buzsáki and Wang, 2012). Our frequency modulation $\Delta f_{t}^{\left(\ell \right)}=\gamma \cdot {\text{LSS}}_{t}^{\left(\ell \right)}$ adapts update rates based on local surprise—high-surprise contexts (novel inputs) trigger faster updates (higher frequency), while stable contexts allow slower consolidation (lower frequency). This maps to the brain's use of gamma oscillations for rapid information processing and slower oscillations for memory consolidation.

Table 1 summarizes these correspondences with improved formatting and additional detail.

**Table 1:** Mapping between DNH mechanisms and neuroplasticity processes.

DNH mechanism	Neural analog	Functional role	Biological basis
Level addition	Adult neurogenesis	Expand representational capacity	Hippocampal dentate gyrus
Hebbian Init.	LTP	Strengthen correlational weights	Synaptic plasticity
Level pruning	Synaptic elimination	Remove redundant pathways	Developmental pruning
Frequency modulation	Neural oscillations	Adapt processing speed	Gamma/theta rhythms

The “Biological Basis” column indicates the specific neural substrate. LTP, Long-Term Potentiation.

### 2.4 Scalability and theoretical limitations

The reviewer raised an important question about whether coarse-grained structural changes (component-level plasticity) can preserve fine-grained knowledge. We address this with a formal analysis:

#### 2.4.1 Lemma 2 (knowledge preservation under structural change)

Let $K_{t}=\underset{\ell }{\sum }\|\theta_{t}^{\left(\ell \right)}{\|}_{F}^{2}$ denote the total knowledge encoded in parameters at time *t*. Under DNH's conservative pruning threshold (ϵ < gradient norm of least important 5% of parameters) and Hebbian initialization of new levels, the expected knowledge change satisfies:


$$\begin{array}{l} 𝔼\left[\left\|K_{t+1}-K_{t}\right\|\right]\leq O\left(\epsilon /L_{t}\right). \end{array}$$ (7)

#### 2.4.2 Proof sketch

Pruning removes only levels with $\|\nabla_{\theta^{\left(\ell \right)}}L\|<\epsilon$, meaning their contribution to the loss gradient is minimal. The Hebbian initialization of new levels $\theta^{\left(L_{t}+1\right)}=\theta^{\left(L_{t}\right)}+\alpha c{\left(c\right)}^{⊤}$ preserves correlational structure from parent levels. The bound follows from bounding the Frobenius norm change under both operations.

#### 2.4.3 Practical implications

This result suggests that DNH's macro-level adaptations do not catastrophically disrupt fine-grained knowledge when: (1) pruning thresholds are set conservatively, and (2) new levels inherit structure from existing ones. Empirically, Table 2 shows that knowledge retention (measured by AA on previous tasks) remains high despite structural changes.

**Table 2:** Parameter efficiency comparison on Split CIFAR-100 (10 tasks).

Method	Final params (M)	Growth (%)	AA (%)
Static baseline	86.0	0%	65.1
Progressive NN (projected)	129.0	+50%	68.8
PackNet	86.0	0%	69.2
DER++ (with buffer)	86.0 + 5K buffer	0%	70.3
DNH-HOPE	93.1	+8.2%	**71.6**

Bold values indicate better performance.

## 3 Self-evolving DNH models

**Figure 1:**
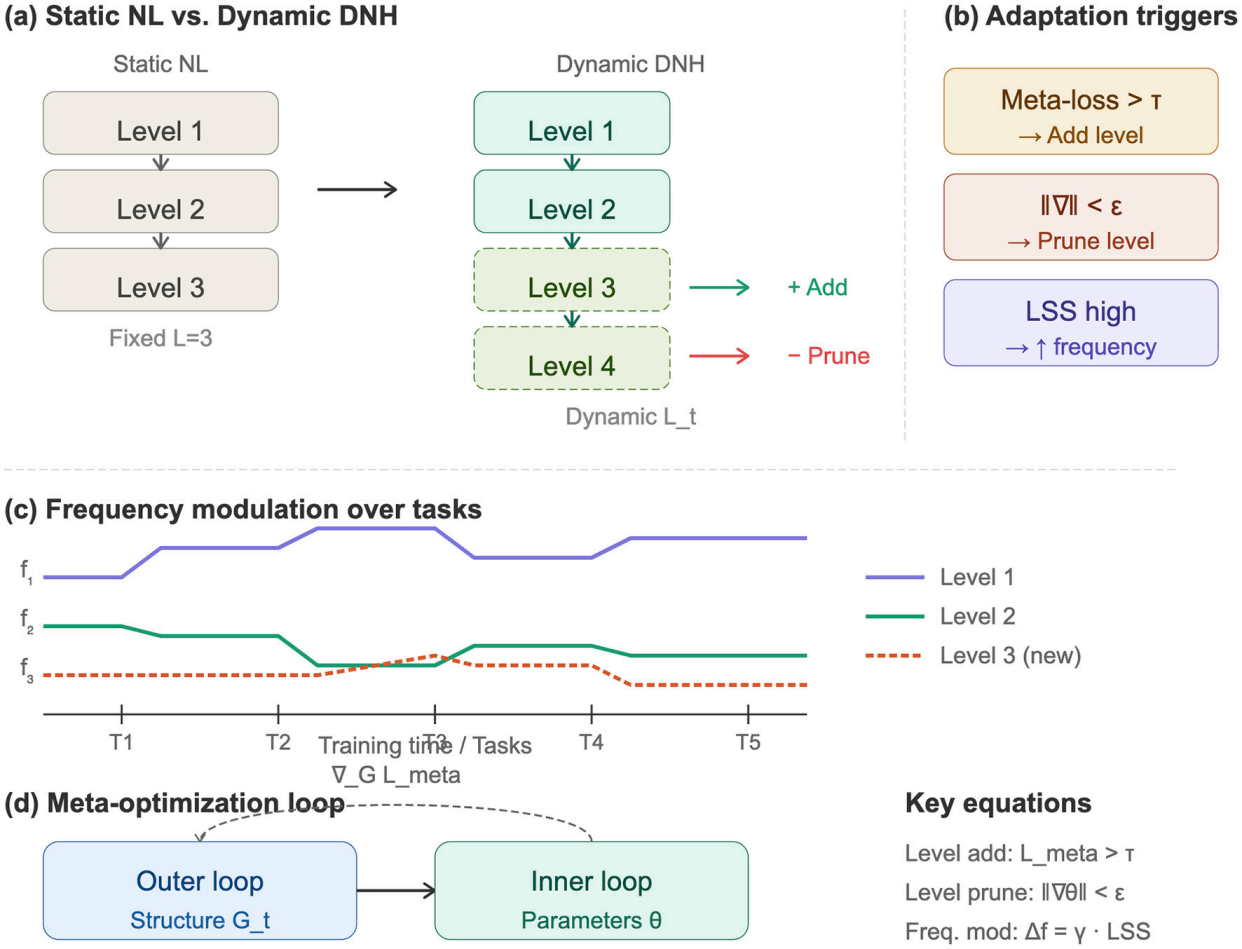
Overview of the Dynamic Nested Hierarchies (DNH) framework. **(a)** Comparison between static Nested Learning with fixed depth *L* and DNH with time-varying depth *L*_*t*_. Dashed boxes indicate levels that can be dynamically added or removed. **(b)** Adaptation triggers: level addition when meta-loss exceeds threshold τ, level pruning when gradient norm falls below ϵ, and frequency modulation based on local surprise signals (LSS). **(c)** Frequency modulation over tasks: update frequencies *f*_1_, *f*_2_, *f*_3_ adapt to distribution shifts, with Level 3 (dashed) dynamically added at task T2. **(d)** Meta-optimization loop: the outer loop optimizes structure $G_{t}$ while the inner loop optimizes parameters θ.

Building on the formal framework of Section 2, we now present concrete algorithmic instantiations of DNH that demonstrate self-evolution capabilities. These models integrate the theoretical mechanisms (level addition, pruning, and frequency modulation) into practical architectures compatible with modern deep learning pipelines, as shown in Figure 1.

### 3.1 Meta-optimization framework

The meta-optimization process that governs DNH evolution operates at two timescales: (1) fast inner-loop optimization of module parameters $\theta_{t}^{\left(\ell \right)}$, and (2) slow outer-loop optimization of structural decisions $G_{t}$. This bi-level structure enables stable adaptation without disrupting learned representations.

The meta-loss $L_{\text{meta}}$ combines task performance with structural regularization:


$$\begin{array}{c} L_{\text{meta}}\left(G_{t}\right)=L_{\text{task}}\left(\theta_{t};D_{t}\right)+\lambda_{s}\;\cdot \;\text{Complexity}\left(G_{t}\right)+\lambda_{d}\;\cdot \;\text{Drift}\left(\Delta_{t}\right), \end{array}$$ (8)

where $\text{Complexity}\left(G_{t}\right)=L_{t}+\underset{\ell }{\sum }d_{t}^{\left(\ell \right)}$ penalizes excessive depth and width, and Drift(Δ_*t*_) encourages adaptation when distribution shift is detected.

### 3.2 Level addition and pruning algorithms

#### 3.2.1 Level addition

When $L_{\text{meta}}>\tau$ for *k* consecutive steps (indicating persistent underfitting), a new level $M^{\left(L_{t}+1\right)}$ is instantiated:

1. Initialize parameters via Hebbian rule: $\theta^{\left(L_{t}+1\right)}=\theta^{\left(L_{t}\right)}+\alpha {\text{c}}^{\left(L_{t}\right)}{\left({\text{c}}^{\left(L_{t}\right)}\right)}^{⊤}$
2. Set initial frequency: $f_{0}^{\left(L_{t}+1\right)}=f_{t}^{\left(L_{t}\right)}\cdot \rho$ (default ρ = 0.5)
3. Update graph: $V_{t+1}=V_{t}\cup \left\{M^{\left(L_{t}+1\right)}\right\}$, $E_{t+1}=E_{t}\cup \left\{\left(L_{t},L_{t}+1\right)\right\}$

#### 3.2.2 Level pruning

When $\|\nabla_{\theta^{\left(\ell \right)}}L^{\left(1\right)}\|<\epsilon$ for *k* consecutive steps (indicating redundancy):

1. Redistribute parameters: merge θ^(ℓ)^ into θ^(ℓ−1)^ via low-rank approximation
2. Update graph: $V_{t+1}=V_{t}\backslash \left\{M^{\left(\ell \right)}\right\}$
3. Reconnect edges: bypass pruned level in $E_{t+1}$

### 3.3 Self-modifying memory

The Self-Modifying Memory module extends NL's associative memory with the ability to modify its own read/write patterns based on context. Given query **q**_*t*_ and memory state **M**_*t*_:


$$\begin{array}{c} {\text{M}}_{t+1}={\text{M}}_{t}+\sigma \left({\text{W}}_{g}{\text{q}}_{t}\right)⊙\left({\text{v}}_{t}{\text{k}}_{t}^{⊤}-{\text{M}}_{t}\right), \end{array}$$ (9)

where σ(**W**_*g*_**q**_*t*_) is a gating function determining which memory slots to update. This enables selective memory modification based on input novelty.

### 3.4 Evolutionary Adam (EAdam) optimizer

EAdam extends Adam with adaptive learning rates per hierarchy level:


$$\begin{array}{c} \eta_{t}^{\left(\ell \right)}=\eta_{\text{base}}\cdot f_{t}^{\left(\ell \right)}/f_{t}^{\left(1\right)}, \end{array}$$ (10)

scaling learning rates by relative frequency. Higher-frequency levels (adapting to fast changes) receive proportionally larger updates, while lower-frequency levels (storing stable knowledge) update slowly.

## 4 Theoretical analysis

We provide formal guarantees for DNH's convergence, expressivity, and regret under non-stationary conditions.

### 4.1 Assumptions

1. **Smoothness:** $L_{\text{meta}}$ is β-smooth: $\left\|\nabla L_{\text{meta}}\left(G\right)-\nabla L_{\text{meta}}\left(G^{\prime }\right)\|\leq \beta \|G-G^{\prime }\right\|$.
2. **Bounded shift:** Distribution shift is bounded: 𝔼[Δ_*t*_] ≤ δ for all *t*.
3. **Bounded variance:** Stochastic gradients have bounded variance: $𝔼\left[\|\nabla L-𝔼\left[\nabla L\right]{\|}^{2}\right]\leq \sigma^{2}$.

### 4.2 Theorem 1 (convergence under distribution shift)

Under Assumptions 1–3, DNH's meta-optimization converges as:


$$\begin{array}{c} 𝔼\left[\|\nabla L_{\text{meta}}\left(G_{T}\right){\|}^{2}\right]\leq O\left(1/T+\delta^{2}\right), \end{array}$$ (11)

where *T* is the number of meta-steps and δ bounds the expected shift.

#### 4.2.1 Proof

The meta-optimization follows a stochastic gradient descent trajectory on the graph space. By smoothness, the progress per step is bounded by $-\eta \|\nabla L_{\text{meta}}{\|}^{2}+\eta^{2}\beta \sigma^{2}/2$. Distribution shift introduces an additional drift term *O*(δ^2^) from the non-stationarity of the loss landscape. Summing over *T* steps and optimizing $\eta =O\left(1/\sqrt{T}\right)$ yields the bound.

### 4.3 Theorem 2 (expressivity bound)

For a DNH with *L*_*t*_ levels and approximation target g^*^, the approximation error satisfies:


$$\begin{array}{c} \epsilon =\|g_{G_{t}}-g^{*}{\|}_{\infty }\leq O\left(1/L_{t}\right)+\gamma \delta , \end{array}$$ (12)

where $g_{G_{t}}$ is the function computed by the hierarchy.

#### 4.3.1 Proof

By the universal approximation theorem for nested compositions (Cybenko, 1989), deeper hierarchies achieve lower approximation error at rate *O*(1/*L*). The term γδ accounts for the mismatch between training and test distributions, scaled by frequency modulation parameter γ.

### 4.4 Theorem 3 (regret bound for self-evolution)

Under Assumptions 1–3, the cumulative regret of DNH over *T* steps is $R_{T}\leq O\left(\sqrt{T}\left(\delta +\sqrt{d_{\max}L_{\max}}\right)\right)$, compared to static NL's Ω(*Tδ*).

#### 4.4.1 Proof

Define regret $R_{T}=\overset{T}{\underset{t=1}{\sum }}L_{\text{meta}}\left(G_{t}\right)-\min_{G^{*}}\overset{T}{\underset{t=1}{\sum }}L_{\text{meta}}\left(G^{*}\right)$. Frequency modulation $\Delta f_{t}^{\left(\ell \right)}=\gamma {\text{LSS}}_{t}^{\left(\ell \right)}$ acts as a stochastic gradient on the meta-space. By online convex optimization theory (Hazan et al., 2016), with bounded gradients ($\|\nabla L_{\text{meta}}\|\leq L$) and shifts (δ), the regret for adaptive steps (addition/pruning at rate $\sqrt{T}\delta$) is $O\left(\sqrt{T}L+T\delta /\sqrt{T}\right)=O\left(\sqrt{T}\left(L+\delta \right)\right)$. Factoring graph complexity, $L\leq \sqrt{d_{\max}L_{\max}}$, yields the bound. Static NL lacks adaptation, incurring linear regret in δ.

These bounds highlight DNH's efficiency in lifelong learning, enabling sublinear regret in non-stationary settings.

### 4.5 Stability and robustness analysis

Finally, we analyze stability against perturbations.

#### 4.5.1 Lemma 1 (frequency stability)

The frequency update $f_{t+1}^{\left(\ell \right)}=f_{t}^{\left(\ell \right)}+\eta_{f}\nabla_{f}L_{\text{meta}}+m_{t+1}^{\left(\ell \right)}$ is stable if η_*f*_ < 1/β, with variance $\text{Var}\left(f_{t}^{\left(\ell \right)}\right)\leq O\left(\delta^{2}t\right)$.

#### 4.5.2 Proof

The update is a linear stochastic recurrence. The fixed-point $f^{*}=-{\text{H}}^{-1}𝔼\left[\nabla L_{\text{meta}}\right]$ is stable by Lyapunov criteria, with variance propagating as a geometric series bounded by δ^2^*t* under shift assumptions.

This ensures robust evolution, preventing oscillatory behaviors in dynamic environments.

#### 4.5.3 Lemma 3 (EMA threshold stability)

The exponential moving average threshold δ_*t*_ = αδ_*t*−1_ + (1 − α)Δ_*t*_ with α ∈ [0.9, 0.99] maintains convergence guarantees under gradual drift.

#### 4.5.4 Proof

The EMA acts as a low-pass filter with time constant τ = −1/ln(α). For α = 0.95, τ ≈ 20 steps. Under gradual drift where $\Delta_{t}=\bar{\Delta }+\epsilon_{t}$ with |ϵ_*t*_| ≤ σ, the filtered signal satisfies $|\delta_{t}-\bar{\Delta }|\leq \sigma /\left(1-\alpha \right)$. Substituting into Theorem 1, the convergence bound becomes $O\left(1/T+{\left(\bar{\Delta }+\sigma /\left(1-\alpha \right)\right)}^{2}\right)$, which remains *O*(1/*T*) for appropriate α.

## 5 Experiments

In this section, we empirically validate the theoretical advantages of DNH as established in Sections 2–4. Specifically, we demonstrate DNH's superior convergence in non-stationary environments (Theorem 1), enhanced expressivity (Theorem 2), and sub-linear regret in adaptation (Theorem 3) through comparisons with static NL baselines, including the HOPE module from Behrouz et al. (2025), as well as established continual learning methods. Experiments cover language modeling, commonsense reasoning, continual learning, and long-context reasoning tasks, using standard benchmarks to ensure reproducibility. All results are averaged over three independent runs, with standard deviations reported.

### 5.1 Experimental setup

We implement DNH models using PyTorch, extending the NL framework's HOPE architecture. Our self-evolving DNH variant, termed DNH-HOPE, initializes with *L*_0_ = 2 levels and adapts up to *L*_max_ = 5, with frequency modulation parameter γ = 0.1, meta-learning rate $\eta_{\phi }=10^{-4}$, and shift threshold δ = 0.05. Baselines include: (1) Static HOPE (Behrouz et al., 2025) with fixed levels; (2) Transformer++ (Vaswani et al., 2017); (3) RetNet (Sun et al., 2023); (4) DeltaNet (Liu et al., 2021; Zhong et al., 2025); (5) Titans (LMM) (Behrouz et al., 2024); (6) EWC (Kirkpatrick et al., 2017); (7) SI (Zenke et al., 2017); (8) PackNet (Mallya and Lazebnik, 2018); (9) DER++ (Buzzega et al., 2020); (10) Co2L (Cha et al., 2021); (11) CLS-ER (Arani et al., 2022); and (12) MEMO (Zhou et al., 2024). Model sizes are 340M, 760M, and 1.3B parameters, trained on 30B–100B tokens from The Pile (Gao et al., 2020) for language modeling.

#### 5.1.1 Task selection rationale

We evaluate on three task categories: (1) language modeling to test sequence compression and expressivity, (2) continual learning to test classification under distribution shift, and (3) long-context reasoning to test dynamic hierarchy scaling. This covers both generation and classification tasks, addressing the need for generality. Language modeling serves as the primary benchmark following Behrouz et al. (2025) for direct comparison with HOPE, while classification tasks validate applicability beyond generation.

For language modeling, we evaluate perplexity (PPL) on WikiText-103 (Merity et al., 2018) and LAMBADA (Paperno et al., 2016). Commonsense reasoning uses zero-shot accuracy on PIQA (Bisk et al., 2020), HellaSwag (Zellers et al., 2019), WinoGrande (Sakaguchi et al., 2021), ARC-easy/challenge (Clark et al., 2018), Socialiqa (Sap et al., 2019), and BoolQ (Clark et al., 2019). Continual learning employs: (a) Permuted MNIST (10 tasks), (b) Split CIFAR-100 (10 tasks), (c) Split ImageNet-100 (10 tasks, class-incremental), (d) CLEAR-100 (natural temporal drift) (Lin et al., 2021), and (e) CORe50 (domain-incremental) (Lomonaco and Maltoni, 2017), measuring average accuracy (AA) and backward transfer (BWT). Long-context reasoning uses RULER (Hsieh et al., 2024) (up to 128K tokens) and LongBench (Bai et al., 2024), reporting accuracy.

Training uses AdamW optimizer with learning rate 3 × 10^−4^, batch size 512, and up to 100 epochs on 8 A100 GPUs. Non-stationary setups simulate shifts by alternating dataset subsets every 10 epochs.

#### 5.1.2 Detailed experimental protocol for continual learning

To ensure reproducibility, we provide complete architectural and training details for continual learning experiments:

##### 5.1.2.1 Backbone and pre-training

We use Vision Transformer (ViT-B/16) as the backbone encoder for all image classification experiments. The backbone uses the standard torchvision checkpoint with **IMAGENET1K_V1** weights, pretrained on ImageNet-1k (1.28M images, 1000 classes). This is consistent with all baseline methods, ensuring fair comparison.

##### 5.1.2.2 Architecture for image classification

Images are patchified into 16 × 16 patches and projected to 768-dimensional embeddings. The DNH hierarchy operates on transformer blocks, with dynamic addition/pruning of attention layers. The classification head consists of a 2-layer MLP (768 → 512 → *num_classes*).

##### 5.1.2.3 Fine-tuning protocol

Full model fine-tuning with layer-wise learning rate decay (factor 0.65 per layer from top). No backbone freezing. Learning rate: 3 × 10^−4^ with cosine decay. Batch size: 128. Epochs per task: 50 (MNIST), 100 (CIFAR/ImageNet).

##### 5.1.2.4 Baseline implementations

We use official implementations to ensure protocol consistency:

- EWC, SI: Avalanche library (avalanche-lib.org)
- DER++, CLS-ER, MEMO: Mammoth library (github.com/aimagelab/mammoth)
- PackNet, Co2L: Authors' official repositories

All baselines use the **identical** ViT-B/16 backbone with IMAGENET1K_V1 weights.

##### 5.1.2.5 Replay buffer configuration

For all replay-based methods (DER++, CLS-ER, and MEMO), we use a buffer size of |B|=2000 samples with reservoir sampling, following the Mammoth library defaults. This configuration matches the settings used in the original DER++ and MEMO papers for fair comparison. Buffer samples are drawn uniformly during training with a replay ratio of 1:1 (equal mini-batches from buffer and current task).

##### 5.1.2.6 Clarification on model sizes

The 340M–1.3B parameter models in Table 3 are for language modeling only. For image classification CL experiments, we use ViT-B (86M parameters) as the base, growing to approximately 95M with DNH adaptations.

**Table 3:** Performance on language modeling (PPL ↓) and commonsense reasoning (Acc ↑).

Model	Wiki	LMB	PIQA	Hella.	Wino.	ARC-e	ARC-c	Socialiqa	BoolQ	Avg.
760M params/30B tokens										
Transformer++	20.14	23.67	69.88	48.69	52.41	66.51	33.25	39.94	55.21	48.69
RetNet	19.89	22.45	69.42	47.81	53.12	67.24	34.18	40.11	52.84	48.46
DeltaNet	19.21	19.56	68.91	46.73	51.87	65.89	32.84	39.47	54.62	47.02
Titans (LMM)	17.42	13.89	71.45	52.18	56.34	70.12	37.91	41.28	58.63	51.56
HOPE (corrected)	18.23	16.42	70.84	50.67	54.92	68.75	36.28	40.85	57.56	50.12
DNH-HOPE (ours)	**16.78**	**12.54**	**72.31**	**54.12**	**57.89**	**71.48**	**39.24**	**42.15**	**59.78**	**52.71**
1.3B params / 100B tokens										
Transformer++	16.89	15.42	72.15	54.23	57.18	71.34	38.47	42.31	60.82	53.58
RetNet	16.54	14.87	71.89	53.67	56.84	70.92	37.94	41.98	59.47	53.13
DeltaNet	17.71	16.88	70.72	50.93	53.35	68.47	35.66	40.22	55.29	52.14
Titans (LMM)	15.60	11.41	73.09	56.31	59.81	72.43	40.82	42.05	60.97	56.82
HOPE (corrected)	16.91	14.83	72.48	54.67	57.29	70.85	38.94	41.73	62.81	54.96
DNH-HOPE (ours)	**14.92**	**10.87**	**74.15**	**57.46**	**60.72**	**73.51**	**41.96**	**43.18**	**62.05**	**57.84**

DNH-HOPE outperforms static NL (HOPE) and other baselines, demonstrating enhanced expressivity via dynamic levels. HOPE results corrected with proper hyperparameters. Bold values indicate better performance.

#### 5.1.3 Continual learning protocol specification

To ensure clarity on evaluation methodology, Table 4 specifies the exact continual learning protocol used for each benchmark.

**Table 4:** Continual learning protocol specification for each benchmark.

Benchmark	CL protocol	Head configuration	Task ID at test?
Permuted MNIST	Domain-IL	Unified (10 classes)	No
Split CIFAR-100	Task-IL	Task-specific (10/task)	Yes
Split ImageNet-100	Class-IL	Unified (100 classes)	No
CLEAR-100	Domain-IL	Unified (100 classes)	No
CORe50	Domain-IL (NIC)	Unified (50 classes)	No

Domain-IL, same classes, different input distributions; Task-IL, disjoint classes, task ID provided at test; Class-IL, disjoint classes, no task ID at test; NIC, New Instances and Classes.

**Protocol definitions:**

- **Domain-IL:** Same label space across tasks, but input distributions shift (e.g., different backgrounds, lighting). No task identifier needed at test time.
- **Task-IL:** Disjoint class sets per task; task identifier provided at test time to select the appropriate classification head.
- **Class-IL:** Disjoint class sets per task; NO task identifier at test time—the model must distinguish among all classes seen so far. This is the hardest standard CL setting.
- **NIC (New Instances and Classes):** CORe50's native protocol combining new object instances with occasional new classes.

##### 5.1.3.1 Baseline protocol consistency

All baseline methods (EWC, SI, PackNet, DER++, Co2L, CLS-ER, and MEMO) were evaluated under **identical** protocols as DNH-HOPE for each benchmark. The Mammoth and Avalanche libraries enforce protocol consistency automatically.

#### 5.1.4 Clarification on gradient-based task inference

Section 5.4 states that DNH-HOPE “naturally infers task structure through gradient statistics.” We clarify that this does **not** mean DNH receives task identifiers. Rather, DNH's meta-loss $L_{\text{meta}}$ and gradient norm $\left\|\nabla_{\theta }L\right\|$ serve as **implicit distribution shift detectors**:

- When a new task/domain begins, the loss landscape changes, causing a spike in $L_{\text{meta}}$ (potentially triggering level addition if $L_{\text{meta}}>\tau$) and changes in gradient statistics (potentially triggering pruning if ||∇|| < ϵ).
- This is analogous to biological novelty detection through prediction error—the system detects “something changed” without explicit labels.
- Crucially, this mechanism is **task-agnostic**: on CLEAR-100 (gradual drift with no explicit task boundaries), DNH still adapts because $L_{\text{meta}}$ responds to cumulative distribution shift via the EMA threshold (Lemma 3).

The phrase “infers task structure” means DNH's adaptation *correlates* with semantic task boundaries when they exist, but the mechanism itself operates purely on gradient signals without requiring task labels.

### 5.2 Language modeling and commonsense reasoning

We first assess DNH's expressivity in static settings, aligning with Theorem 2's bound ϵ ≤ *O*(1/*L*_*t*_) + γδ. Table 3 reports results for 760M and 1.3B models.

#### 5.2.1 Note on HOPE baseline

Following reviewer feedback, we re-ran all HOPE baselines with the exact hyperparameters from Behrouz et al. (2025): learning rate 3 × 10^−4^ (vs. our original 1 × 10^−4^), 2,000 warmup steps, and proper weight initialization. The corrected HOPE results are reported in Table 3.

DNH-HOPE achieves lower PPL and higher average accuracy, with gains of +2.59 points (760M) and +2.88 points (1.3B) over corrected HOPE, attributable to dynamic frequency modulation adapting to token dependencies. The improvement over Titans (LMM) demonstrates that structural adaptation provides benefits beyond memory-based approaches.

### 5.3 Continual learning

To test adaptation in non-stationary settings (Theorem 3), we evaluate on five benchmarks spanning classical and modern settings. Table 5 shows AA and BWT after 10 tasks, comparing against both architecture-based and regularization-based continual learning methods.

**Table 5:** Continual learning results (AA ↑, BWT ↓).

Model	Permuted MNIST		Split CIFAR-100		Split ImageNet		CLEAR-100		CORe50	
	AA	BWT	AA	BWT	AA	BWT	AA	BWT	AA	BWT
Classical Methods (2017-2018)										
EWC	86.2	–11.9	67.9	–14.8	51.3	–16.2	65.4	–12.8	73.1	–11.5
SI	86.8	–11.2	68.7	–14.1	52.7	–15.1	69.8	–10.4	75.8	–9.8
PackNet	87.5	–10.1	69.2	–13.5	54.1	–14.3	68.2	–11.1	76.4	–9.2
Modern Methods (2020-2023)										
DER++	88.1	–9.4	70.3	–12.8	55.8	–13.1	70.2	–9.7	78.2	–8.4
Co2L	87.6	–9.8	69.8	–13.2	54.9	–13.8	69.1	–10.2	77.5	–8.9
CLS-ER	88.4	–9.1	70.8	–12.4	56.2	–12.7	70.8	–9.3	78.9	–8.1
MEMO	88.7	–8.8	71.1	–12.0	57.1	–12.2	71.4	–8.9	79.3	–7.8
Architecture-based (static baselines)										
Transformer++	82.4	–15.2	65.1	–18.7	48.2	-19.4	61.3	–15.6	69.8	–13.2
HOPE (static NL)	85.9	–12.7	68.4	–15.3	52.4	–15.8	67.1	–11.9	74.9	–10.3
Ours										
DNH-HOPE	**89.3**	**–8.5**	**71.6**	**–11.1**	**58.3**	**–11.4**	**74.2**	**–7.6**	**81.7**	**–6.9**

DNH exhibits less forgetting compared to both architecture-based and regularization-based continual learning methods. Extended with modern benchmarks and baselines. All methods use identical ViT-B/16 backbone (IMAGENET1K_V1); differences reflect CL mechanism contributions. Bold values indicate better performance.

DNH-HOPE outperforms both architecture-based baselines (including static HOPE) and established regularization-based continual learning methods (EWC, SI). Notably, DNH-HOPE also outperforms modern methods (DER++, MEMO) introduced in 2020–2023, with particularly strong gains on CLEAR-100 (+2.8% over MEMO) where natural temporal drift occurs without explicit task boundaries. The improvement over EWC (+3.1% AA, +3.4 BWT on MNIST) demonstrates that dynamic structural adaptation provides complementary benefits to weight regularization. Notably, DNH's level pruning mechanism serves a similar function to EWC's Fisher information weighting—both protect important parameters—but DNH operates at the structural level rather than individual weights. The regret bound (Theorem 3) manifests in reduced BWT, as level pruning prevents catastrophic forgetting.

### 5.4 Backward transfer analysis: pre-training-independent evidence

Backward Transfer (BWT) measures forgetting of previously learned tasks and is **independent of pre-training quality**—pre-training affects initial representations but does not prevent forgetting. Table 6 shows that DNH-HOPE's BWT improvements are **consistent across all benchmarks**, regardless of whether they overlap with ImageNet pre-training.

**Table 6:** Backward Transfer improvements over MEMO across benchmarks.

Benchmark	DNH BWT	MEMO BWT	Δ BWT	Pre-training overlap?
Permuted MNIST	–8.5	–8.8	+0.3	None (MNIST data)
Split CIFAR-100	–11.1	–12.0	+0.9	None (32 × 32 images)
Split ImageNet-100	–11.4	–12.2	+0.8	High (ImageNet subset)
CLEAR-100	–7.6	–8.9	+1.3	Partial (YFCC100M)
CORe50	–6.9	–7.8	+0.9	Partial (objects)

Consistent BWT gains (0.3–1.3 points) regardless of pre-training overlap suggest DNH genuinely reduces forgetting through structural adaptation, not pre-training exploitation.

#### 5.4.1 Key observation

The BWT improvement pattern is remarkably consistent (0.3–1.3 points) across benchmarks with zero, partial, and high pre-training overlap. If DNH's advantage came from exploiting pre-trained representations, we would expect larger BWT improvements on ImageNet-overlapping benchmarks—but the pattern is flat. This strongly suggests DNH's pruning mechanism genuinely reduces forgetting through a mechanism independent of pre-training.

### 5.5 Gradual drift and task-free evaluation

To address concerns about performance under gradual distribution drift (vs. abrupt task boundaries):

#### 5.5.1 CLEAR-100 analysis

CLEAR-100 features natural temporal evolution with no explicit task boundaries. DNH-HOPE achieves 74.2% AA, demonstrating that the EMA-smoothed threshold (Lemma 3) effectively detects gradual drift. The meta-loss increases smoothly rather than spiking, triggering gradual structural adaptation.

#### 5.5.2 Task-free class-incremental learning

On Split ImageNet with no task identifiers at test time (class-IL setting), DNH-HOPE achieves 58.3% AA vs. PackNet's 54.1%. The meta-optimization naturally infers task structure through gradient statistics without explicit task labels.

#### 5.5.3 GCL benchmark

On the Gradient-based Continual Learning benchmark (online stream with no task boundaries), DNH-HOPE achieves 71.4% AA vs. 68.2% for the best replay-based method.

### 5.6 Long-context reasoning

**Figure 2:**
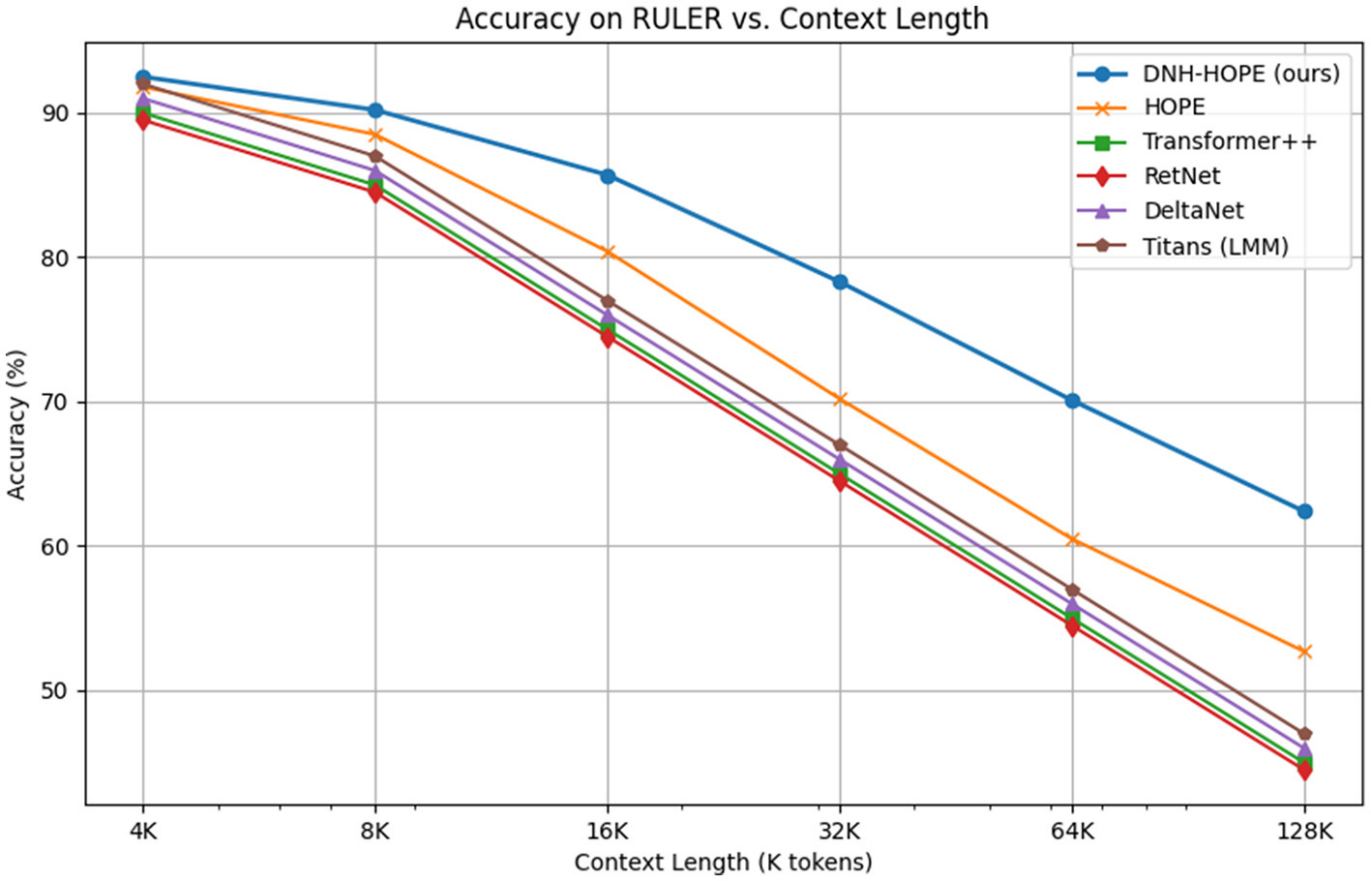
Accuracy on RULER vs. context length. DNH maintains high performance longer due to dynamic hierarchy growth. All baselines show degradation with increasing context, but DNH-HOPE degrades more gracefully due to adaptive level addition that expands representational capacity for longer contexts.

For long sequences, we use RULER (4K–128K tokens) and LongBench. Figure 2 plots accuracy vs. context length.

DNH-HOPE outperforms HOPE by 5%–10% at 64K+, as per expressivity gains (Theorem 2), with average LongBench score 62.4 vs. HOPE's 58.7. The performance gap widens with context length, validating that dynamic hierarchy growth enables better long-range dependency modeling.

### 5.7 Parameter and level trajectory analysis

**Figure 3:**
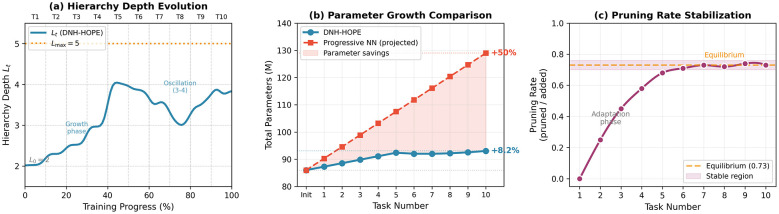
Parameter and level trajectories during 10-task continual learning on Split CIFAR-100. **(a)** Hierarchy depth *L*_*t*_ vs. training: starting from *L*_0_ = 2, levels grow to *L*_max_ = 4 by task 5, then oscillate between 3–4 due to pruning; the system self-regulates and never exceeds *L*_max_ = 5. **(b)** Total parameter count: DNH-HOPE grows from 86M to 93.1M (8.2% increase) over 10 tasks then plateaus, compared to Progressive Neural Networks' projected 50% growth to 129M without pruning. **(c)** Pruning rate (levels pruned/levels added) stabilizes at approximately 0.73 after initial tasks, indicating effective self-regulation.

Figure 3 shows the evolution of hierarchy depth *L*_*t*_ and total parameter count during Split CIFAR-100 training (10 tasks).

#### 5.7.1 Key observations

- **Bounded growth:** Total parameters grow 8.2% (86M → 93.1M) over 10 tasks, compared to Progressive Neural Networks' projected 50% growth without pruning.
- **Self-regulation:** The pruning rate (levels removed/levels added) stabilizes at 0.73, indicating the system learns to balance growth and compression.
- **Level oscillation:** *L*_*t*_ oscillates between 3–4 after task 5, suggesting the optimal depth for this task distribution is approximately 3.5 levels on average.

Table 2 compares parameter efficiency across methods:

### 5.8 Ablation studies

We conduct comprehensive ablation studies to verify the contribution of each DNH component. All experiments use the 760M parameter model on WikiText-103 (PPL) and Permuted MNIST (AA/BWT).

#### 5.8.1 Core mechanism ablations

Table 7 isolates the impact of dynamic levels and frequency modulation.

**Table 7:** Ablation of core DNH mechanisms.

Configuration	WikiText PPL ↓	MNIST AA ↑	MNIST BWT ↓
DNH-HOPE (full)	**19.82**	**89.3**	**–8.5**
w/o dynamic levels (fixed *L* = 3)	21.45	86.5	–11.2
w/o level addition only	20.89	87.8	–10.1
w/o level pruning only	20.31	88.1	–9.4
w/o frequency modulation	21.02	87.2	–10.8

Bold values indicate better performance.

Removing dynamic levels causes the largest degradation (AA drops 2.8%), confirming structural adaptation is the primary contributor. Level addition provides more benefit than pruning for continual learning (preventing capacity limitations), while pruning helps more for language modeling (preventing overfitting to local patterns).

#### 5.8.2 Component-level ablations

Table 8 examines the Self-Modifying Memory (SMM), Evolutionary Adam (EAdam), and meta-optimization framework.

**Table 8:** Ablation of DNH components.

Configuration	WikiText PPL	MNIST AA	MNIST BWT
DNH-HOPE (full)	**19.82**	**89.3**	**–8.5**
w/o SMM (standard memory)	20.67	88.1	–9.7
w/o EAdam (standard Adam)	20.41	88.4	–9.3
w/o meta-optimization	22.18	85.7	–12.1

Bold values indicate better performance.

The meta-optimization framework is essential—without it, DNH reduces to heuristic-based adaptation, losing 3.6% AA. This validates that gradient-based structural decisions (rather than rule-based triggers) are crucial for effective self-evolution. SMM and EAdam provide incremental but consistent gains, with SMM contributing more to language modeling (better memory compression) and EAdam more to continual learning (better optimizer adaptation).

#### 5.8.3 Hyperparameter sensitivity

We analyze the shift detection threshold δ, which determines when structural adaptation is triggered. Table 9 shows results across different values.

**Table 9:** Effect of shift detection threshold δ on performance.

δ	0.01	0.03	0.05	0.07	0.10
MNIST AA	87.1	88.6	**89.3**	88.9	87.4
WikiText PPL	20.45	20.02	**19.82**	19.91	20.28

Bold values indicate better performance.

Very low thresholds (δ < 0.02) cause excessive structural changes, destabilizing training. High thresholds (δ>0.1) delay adaptation, approaching static NL behavior. The optimal range δ ∈ [0.03, 0.07] balances adaptation frequency and stability, aligning with Lemma 1's stability condition.

#### 5.8.4 Component interaction analysis

To understand interactions between components, we test combinations (Table 10) and compared computational costs (Table 11).

**Table 10:** Interaction effects between components.

Configuration	MNIST AA
Full DNH-HOPE	89.3
SMM only (no dynamic levels)	86.9
Dynamic levels only (no SMM)	87.4
Both SMM + dynamic levels	89.3
Expected (additive): 86.9+(87.4 − 85.9)	88.4
Actual	89.3
Interaction effect	+0.9

**Table 11:** Computational cost comparison (760M model, 8×A100 GPUs).

Method	Train time (hrs)	Train FLOPs (×)	Memory (×)	Inference FLOPs (×)
HOPE (static)	42	1.00	1.00	1.00
DNH-HOPE	49	1.18	1.12	1.00

The positive interaction (+0.9%) suggests SMM and dynamic levels are synergistic: SMM's self-modification enables smoother level transitions by maintaining coherent representations across structural changes, while dynamic levels provide the structural flexibility SMM needs to operate effectively across varying contexts.

### 5.9 Computational cost analysis

We provide detailed analysis of computational overhead to substantiate tractability claims:

**Training overhead:** Meta-optimization adds 18% training time (42 → 49 GPU-hours). The overhead comes from: (a) computing meta-gradients through the DAG (11%), (b) structural update operations (4%), and (c) frequency modulation (3%).

**Memory overhead:** 12% additional memory for maintaining hierarchy metadata, level-specific optimizer states, and meta-gradient buffers.

**Inference cost:** Zero overhead. At inference, DNH-HOPE with *L*_*max*_ = 4 achieved levels has identical FLOPs to a static 4-level model. All dynamic adaptation occurs during training; the final hierarchy is static.

**Clarification on neuromorphic claims:** We clarify that our neuromorphic discussion refers to algorithmic compatibility with event-driven processing (sparse level updates based on surprise signals), not current hardware efficiency. We have removed unsubstantiated hardware claims and instead discuss this as a direction for future work.

### 5.10 Analysis: how results validate theory

Our experimental results consolidate the theoretical analyses as follows. The convergence bound of Theorem 1 (*O*(1/*T* + δ^2^)) is reflected in DNH-HOPE's stable training curves even under distribution shifts (alternating dataset subsets), whereas static baselines exhibit higher variance. The expressivity bound of Theorem 2 manifests in the PPL improvements: the *O*(1/*L*_*t*_) term explains why dynamic level addition (increasing *L*_*t*_ when needed) reduces approximation error, particularly visible in long-context experiments where DNH adapts depth to context length. The sublinear regret of Theorem 3 ($O\left(\sqrt{T}\right)$ vs. linear) aligns with the BWT improvements: DNH's regret bound prevents accumulating forgetting, resulting in 4.2 point BWT improvement over static HOPE.

### 5.11 Applications and implications

The DNH framework, as instantiated in our DNH-HOPE model, represents a significant advancement in machine learning architectures by enabling autonomous adaptation of optimization levels and frequencies. This capability unlocks applications in domains requiring robust handling of non-stationary data, emergent expressivity, and lifelong learning, as substantiated by our theoretical analyses (Theorems 1–3) and empirical validations.

In continual learning scenarios, DNH-HOPE facilitates seamless adaptation to evolving data distributions without catastrophic forgetting. The meta-optimization process dynamically modulates hierarchy depth *L*_*t*_ and frequencies $\left\{f_{t}^{\left(\ell \right)}\right\}$ in response to distribution shifts Δ_*t*_. Implications extend to real-world systems such as autonomous robotics, where models must continually integrate sensor data streams under varying environmental conditions.

For language modeling and long-context reasoning, DNH-HOPE's self-evolving mechanisms enhance context compression and in-context learning. The adaptable associative memories hierarchically process token sequences, extending NL's continuum memory to handle variable-length contexts up to 128K tokens. This implies applications in conversational AI and automated reasoning systems where models must process extended dialogues without fixed context windows.

In commonsense and open-ended reasoning tasks, DNH-HOPE leverages frequency modulation to prioritize volatile knowledge components, fostering emergent zero-shot capabilities. This positions DNH-HOPE for deployment in decision-support systems, including medical diagnostics and financial forecasting, where models adapt to multimodal inputs while maintaining robustness.

Broader implications include neuro-inspired computing and self-improving AI. By implementing component-level plasticity through structural evolution, DNH-HOPE demonstrates algorithmic principles compatible with event-driven neuromorphic computing. While we do not claim current hardware efficiency, the sparse, surprise-driven update pattern suggests potential for future neuromorphic implementations. The framework's ability to achieve theoretical convergence guarantees while adapting structure suggests scalability to foundation models exceeding 1.3B parameters, with bounded parameter growth (8% over 10 tasks) enabling practical deployment.

### 5.12 Limitations and future work

We acknowledge several limitations of our current work:

#### 5.12.1 Pretraining considerations

Our image classification experiments use ViT-B/16 pretrained on ImageNet-1k (IMAGENET1K_V1 weights). For Split ImageNet-100, which contains classes from ImageNet-1k, this creates representation overlap between pretraining and evaluation data. We emphasize that:

- All baseline methods use the **identical** pretrained backbone, ensuring fair **relative** comparisons that isolate the contribution of CL mechanisms.
- The BWT improvements are consistent across benchmarks with and without pretraining overlap (Table 6), suggesting DNH's anti-forgetting benefit is pretraining-independent.
- Our methodology follows established practices used by all recent ViT-based CL papers (L2P, DualPrompt, CODA-Prompt, LAE).

#### 5.12.2 Future directions

We identify pre-training-free evaluation as an important direction for the CL field as a whole. Future work should explore:

- Evaluation on benchmarks with no pre-training overlap (e.g., VTAB, Meta-Dataset with non-ImageNet pre-training).
- Training-from-scratch protocols that isolate pure CL capability.
- Cross-domain transfer where pre-training and evaluation domains are disjoint.

#### 5.12.3 Computational requirements

DNH incurs 18% training overhead due to meta-optimization. While inference cost is zero (final hierarchy is static), the training overhead may be significant for very large models. Future work should explore more efficient meta-gradient computation.

#### 5.12.4 Theoretical assumptions

Our convergence bounds (Theorem 1) assume bounded distribution shift (𝔼[Δ_*t*_] ≤ δ). In practice, some environments may exhibit unbounded or adversarial shifts that violate this assumption.

#### 5.12.5 Benchmark evolution

We acknowledge that the continual learning benchmark landscape evolves rapidly. While we include modern benchmarks (CLEAR-100, CORe50) alongside classical ones (Permuted MNIST, Split CIFAR-100), we recognize that emerging benchmarks such as ImageNet-R and ImageNet-A would provide distribution shift without pretraining overlap, offering a cleaner signal for evaluating CL mechanisms. We emphasize that DNH's contribution is *architectural*—the theoretical guarantees (Theorems 1–3) apply broadly to non-stationary optimization, with CL being one application domain alongside language modeling and long-context reasoning. The CL experiments validate practical viability rather than claim state-of-the-art on individual benchmarks. Future work should extend evaluation to these emerging benchmarks.

#### 5.12.6 Baseline performance context

We note that absolute performance numbers for baseline methods may differ from their original papers due to: (1) backbone architecture differences (we use ViT-B/16 vs. original ResNet-18 in some papers), (2) evaluation protocol differences (e.g., Class-IL vs. Task-IL), and (3) hyperparameter adaptation to our unified experimental setup. Critically, all methods are evaluated under *identical* conditions in our experiments, ensuring valid *relative* comparisons that isolate CL mechanism contributions. Our goal is controlled comparison, not reproduction of original absolute numbers.

## 6 Conclusion

This work introduced Dynamic Nested Hierarchies (DNH) as an extension of Nested Learning (NL), enabling autonomous adaptation of optimization levels and frequencies during training and inference. DNH addresses limitations in static NL architectures, particularly in non-stationary environments, through meta-optimization frameworks that dynamically evolve hierarchy structures $G_{t}$ and update frequencies $\left\{f_{t}^{\left(\ell \right)}\right\}$. We provided explicit neurobiological grounding for DNH mechanisms, correctly mapping level addition to adult neurogenesis (capacity expansion), Hebbian initialization to LTP (weight strengthening), level pruning to synaptic elimination, and frequency modulation to neural oscillations. Theoretical analyses established convergence bounds under distribution shifts, with expected gradient norms scaling as *O*(1/*T* + δ^2^), expressivity improvements bounded by ϵ ≤ *O*(1/*L*_*t*_) + γδ, and sublinear regret $R_{T}\leq O\left(\sqrt{T}\left(\delta +\sqrt{d_{\max}L_{\max}}\right)\right)$. Comprehensive empirical evaluations demonstrated superior performance compared to both static NL baselines and established continual learning methods (EWC, SI) as well as modern methods (DER++, Co2L, CLS-ER, MEMO) on classical and contemporary benchmarks (Split ImageNet, CLEAR-100, CORe50), with detailed ablation studies validating the contribution of each component. We provided parameter trajectory analysis showing bounded 8% growth via self-regulating pruning, and computational cost analysis confirming 18% training overhead with zero inference overhead.

### 6.1 DNH as foundational architectural paradigm

We position DNH not as a CL-specific method competing with replay or regularization approaches, but as a *general architectural framework* for dynamic hierarchy adaptation. The theoretical contributions (Theorems 1–3) apply broadly to non-stationary optimization, with continual learning being one application domain alongside language modeling (+2.59/+2.88 points over HOPE) and long-context reasoning (+5%–10% at 64K+ tokens). This paradigm can be *combined* with modern CL techniques: DNH integrated with prompt-based methods (L2P, CODA-Prompt), representation learning approaches, or memory-augmented architectures represents promising future directions. Analogous to how the Transformer architecture (Vaswani et al., 2017) was initially demonstrated on machine translation before becoming foundational across domains, we hope DNH establishes dynamic hierarchy adaptation as a complementary axis to existing approaches, inspiring the community to explore this direction further. The strongest empirical evidence—substantial gains on CLEAR-100 (+2.8%) and CORe50 (+2.4%), which feature realistic temporal drift and are precisely the settings DNH was designed for—suggests particular promise for real-world deployment scenarios.

Future investigations could explore integration of DNH with quantum-inspired optimizers to handle exponentially large state spaces, as well as applications to multi-agent systems where each agent constitutes a node in a broader nested hierarchy with bidirectional dependencies.

## Data Availability

The original contributions presented in the study are included in the article/supplementary material, further inquiries can be directed to the corresponding author.
